# Destination memory: Memory associated with social interactions

**DOI:** 10.3389/fpsyg.2022.1061275

**Published:** 2023-02-20

**Authors:** Mohamad El Haj

**Affiliations:** ^1^Laboratoire de Psychologie des Pays de la Loire (LPPL-EA 4638), Nantes Université, Université d'Angers, Nantes, France; ^2^CHU Nantes, Clinical Gerontology Department, Nantes, France; ^3^Institut Universitaire de France, Paris, France

**Keywords:** communication, destination memory, memory, social cognition, episodic memory

## Abstract

Within the field of memory research, studies on destination memory (e.g., the ability to remember to whom information was previously told) show how it is closely associated with social cognition. The present review thus summarizes the literature on destination memory and demonstrates how it involves social interaction. It offers a comprehensive picture of the many factors that may influence destination memory and distinguishes factors related to the recipient (e.g., familiarity, emotional states, and distinctiveness/attractiveness) and sender of information (e.g., the sender’s extroversion) in social communications. It suggests that destination memory involves the ability of the sender to infer the cognitive/affective state of the recipient and to attribute the output message to a recipient-related stereotype. Extrovert senders may also easily remember the destination as they typically value social communication, public sharing and processing of social information. Destination memory also involves features such as familiarity, age, emotional state, distinctiveness, and attractiveness of the recipient. By offering a comprehensive framework of how destination memory functions in everyday life interactions, the present review shows how destination memory is intimately associated with communicative efficacy and social interactions.

## 1. Introduction

Imagine yourself in the following situations: on a lunch break, you are telling your colleagues about your last conference abroad, and then realize that you have already told them about it; or you are telling a joke to a friend who does not laugh as he has already heard that joke from you. These embarrassing situations illustrate failures of destination memory. Destination memory, or the ability to remember to whom information was previously told (Gopie and Macleod, 2009; Gopie et al., 2010), is intimately associated with social communication. Destination memory influences communicative efficacy and social interactions. Failures of destination memory result in redundancy, i.e., the tendency to repeat the same information to the same recipient, which hinders social communication especially in patients with amnesia.

Research has identified the cognitive processes linking this memory system to episodic memory and the social cognition processes that shape destination memory. Studies have assessed both the function and dysfunction of destination memory, especially in patients with amnesia. The present review examines the relationship between destination memory, episodic memory, and social cognition. It offers a comprehensive picture of the many factors that may influence destination memory, with a distinction between factors that are related to the recipient (e.g., familiarity, emotional states, distinctiveness/attractiveness) and those that are related to the sender of information (e.g., the sender’s extroversion) during social communications. However, to comply with the objective of this review, the next section places destination memory in the context of the episodic memory system.

## 2. Destination memory: A component of episodic memory?

One of the early preoccupations of research on destination memory was its status compared to that of episodic memory (El Haj et al., 2013, 2015b), probably since rooting destination memory into a wider memory system would establish the cognitive foundation of its study. In this attempt, research identified three main cognitive mechanisms that are involved in both destination memory and episodic memory: context recall, binding, and subjective reliving. Regarding context recall, episodic memory is typically defined as the ability to remember information about the context in which a unique episode took place (Tulving, 2002). This context recall was also found to be involved in destination memory, a link assessed in a study asking participants to tell information (e.g., Madrid is the capital of Spain) to either colored or black and white photographs of celebrities (e.g., Barack Obama; El Haj and Allain, 2014). In a subsequent recognition test, participants had to decide whether they had told that information to these faces or not. Participants were also asked to remember the context of encoding, particularly whether the photographs were presented in color or in black and white. Analysis demonstrated that destination memory was predicted by context recall.

As stated above, destination memory relies on the ability to remember the context in which information was previously told. It involves binding, i.e., the ability to associate an event with its context of acquisition (Mitchell et al., 2000a; Parra et al., 2010; Shimamura, 2010; Kessels and Kopelman, 2012; El Haj, 2016; Johnson and Jefferson, 2018). This hypothesis was proposed in a study in which patients with amnesia (i.e., patients with Korsakoff’s syndrome) and control participants were assessed in a destination memory task and a binding task (El Haj et al., 2016b). In the binding task, participants were asked to remember the location of letters that were exposed in various grids, and to decide in a later recognition test whether the letters appeared in the same location as before. Results showed that destination memory performances were predicted by binding. Similar findings were reported by studies on normal aging (El Haj and Allain, 2014) and schizophrenia (El Haj et al., 2017), mirroring research demonstrating how deficits in associative processes (i.e., binding) hinder episodic memory in general (Chalfonte et al., 1996; Mitchell et al., 2000b; Naveh-Benjamin et al., 2003; Provyn et al., 2007). Destination memory requires the ability to associate an event (i.e., the output message) with its context of acquisition (i.e., to whom) to form an integrated episode. Binding may thus underlie the relationship between destination memory and episodic memory by facilitating the successful association between output information and its appropriate recipient.

This association probably contributes to the uniqueness of the destination episode and to our ability to mentally travel along a time continuum to re-experience the encoding event (i.e., to subjectively re-experience that moment when we told that piece of information to that person). The involvement of the subjective experience in destination memory was highlighted in a study in which patients with amnesia (i.e., Alzheimer’s disease) and control participants were asked to tell information to photographs of celebrities (El Haj et al., 2014b). In a subsequent recognition test, participants had to decide whether they had previously told that fact to that face. In addition to this typical destination memory task, participants were asked whether they could mentally project themselves in time to remember the fact–face pairs. Results showed significant positive correlations between destination memory and mental time travel. Similar findings were reported by a study on patients with Huntington’s disease (El Haj et al., 2016a).

Altogether, destination memory can be embedded into the episodic memory system (El Haj et al., 2014a; El Haj and Miller, 2018a,b; Wasserman et al., 2020). Like episodic memory, destination memory allows the reconstruction of the context in which an event has occurred (here, to whom information was previously told). This context reconstruction contributes to the uniqueness of an encoded event (i.e., that moment when I told that piece of information to that person) and to the ability to travel along a time continuum to re-experience that event. Although destination memory can be embedded into the larger episodic memory system, it can be also embedded into a social cognition framework, as explained in the following section explains.

## 3. Destination memory: The social memory

While destination memory may be considered as a component of the episodic system, it is special as it involves social interaction. The relationship between destination memory and social cognition has been shown in the literature. Although this literature has demonstrated how destination memory can be influenced by several social factors (e.g., familiarity, emotion, distinctiveness/attractiveness), it has not attempted to describe the relationship between these processes or to classify them. To bridge this gap, the present review offers a comprehensive picture of the multitude of factors that may influence destination memory. Since destination memory involves both the recipient of the information and its sender, this research suggests a basic distinction between recipient-related (e.g., familiarity, emotional states, distinctiveness/attractiveness) and sender-related factors (e.g., extroversion). As explained below, destination memory may be studied by focusing on features related to the recipient such as familiarity, age, and emotional states, distinctiveness, and attractiveness of the recipient. It may also be studied by analyzing features related to the sender, such as his/her ability to infer the mental state of the recipient, the stereotypes attributed by the sender to the destination, as well as his/her extroversion and the effect of self-related information.

### 3.1. How the recipient’s characteristics influence destination memory

In everyday life, we tend to communicate with familiar rather than with unfamiliar people. This familiarity issue was investigated in a study in which participants were asked to tell information to photographs of famous faces or to unfamiliar faces (El Haj et al., 2015d). In a subsequent recognition test, participants were asked to decide whether they had previously told that piece of information to that face. Results demonstrated higher destination memory for familiar than for unfamiliar faces. Although familiarity can be considered as a main feature facilitating destination memory, other basic features of the interlocutors may also influence destination memory, such as their age. A study has assessed whether older adults might show better destination memory for older interlocutors than for younger ones (El Haj et al., 2018a). Older and younger adults were asked to tell information to older and younger destinations. In a subsequent recognition test, they were asked to remember whether they had previously told the information to an older or younger destination. Results demonstrated higher memory for older than for younger destinations in older adults and the opposite pattern was observed in younger adults. These findings suggest how the age of the recipient can play a crucial role in our ability to remember to whom information was previously told. While the recipient’s age can influence retrieval, his/her gender does not necessarily have the same effect, as shown by a study on gender differences in destination memory (El Haj et al., 2020). Female and male participants were asked to tell information to pictures depicting female and male faces. In a subsequent recognition test, participants had to decide to whom they had told the information. While analysis demonstrated better destination memory (regardless of the destination’s gender) in female participants than in male participants, both female and male participants demonstrated similar memory for female and male destinations. The gender of the recipient thus seems to play no significant role in retention.

So far, our research has shown how destination memory can be influenced by the familiarity and age of the recipient. Other characteristics of the recipient such as his/her emotional states, distinctiveness, and attractiveness may also have an impact. A study investigated whether the receiver’s emotional states may enhance destination memory in normal aging (El Haj et al., 2015a). Older and younger adults were asked to tell neutral information to neutral, positive or negative destinations. Results demonstrated higher memory for negative than for positive destinations, and higher memory for positive than for neutral destinations in older adults. However, the younger adults demonstrated similar memory for both neutral and emotional destinations. Unlike younger adults, older adults therefore place higher emphasis on emotional faces than on neutral ones, exhibiting better memory for emotional than for neutral destinations. Similar findings were reported in a study demonstrating higher memory for emotional than for neutral destinations in older adults, but not in patients with Alzheimer’s disease (El Haj et al., 2015e).

Like emotional states, the recipient’s distinctiveness can influence destination memory. A study investigated whether the existence of distinctive features of the recipient (e.g., tattoos) might improve destination memory. It showed higher destination memory for recipients with distinctive features compared to those with none (Barros et al., 2021). These findings highlight how, when remembering to whom information was previously told, we may easily attribute the information to its appropriate recipient when the latter has a distinctive feature. The same can be said of attractiveness. In another study, participants were asked to tell information to attractive faces, to unattractive ones or to neither-attractive-nor-unattractive faces (El Haj and Ndobo, 2021). Results demonstrated higher memory for attractive than for neither-attractive-nor-unattractive recipients, and higher memory for unattractive than for neither-attractive-nor-unattractive recipients. Both attractive and unattractive recipients thus influence destination memory, probably because both types are distinctive. This assumption was supported by the U-shaped model of Ellis et al. (1973), which accounts for the relationship between facial recognition and attractiveness. According to this model, faces of average attractiveness are the least distinctive and consequently more difficult to remember than very attractive or very unattractive faces, which are more distinctive and therefore provide more cues for retrieval.

Altogether, this section has shown how destination memory can be influenced by recipient-related features, such as familiarity, age, emotional states, distinctiveness and attractiveness. However, destination memory can also be influenced by features related to the sender, as discussed in the next section.

### 3.2. How the sender’s characteristics influence destination memory

In addition to the recipient’s features, destination memory can also be influenced by characteristics related to the sender, such as his/her ability to infer the mental state of the recipient (i.e., theory of mind), the stereotypes he/she attributes to the recipient, as well as his/her extroversion and the effect of self-related information. To begin with the theory of mind or the ability to process the cognitive and affective attributes of others (Shamay-Tsoory and Aharon-Peretz, 2007), destination memory can be related to this theory, as individuals adjust the content of our output messages to recipients by observing and evaluating their feedback. In a study on the relationship between destination memory and theory of mind (El Haj et al., 2016c), participants were asked to remember to whom information had been told previously. In addition to the typical destination memory test, participants performed theory of mind tests. Results demonstrated that destination memory was predicted by performances in theory of mind tests. Another study extended these findings to amnesia by demonstrating that decline in destination memory in patients with Alzheimer’s disease was predicted by decline in theory of mind (El Haj et al., 2015c). The relationship between destination memory and theory of mind may be attributed to the fact that theory of mind allows deep processing and efficient association between information and its recipient (Haj et al., 2019). Theory of mind may even mediate the ability to deceive when telling information to a given recipient. This assumption is supported by research demonstrating that when deceiving a given destination, the ability to infer and monitor the cognitive states, thoughts and intentions of the recipient allows a better association between information and destination (El Haj et al., 2018b).

In addition to the ability of the sender to infer the cognitive and affective states of the recipient, destination memory can also be influenced by stereotypes attributed by the sender to the recipient. The effect of stereotypes on destination memory was investigated in a study in which participants were asked to tell facts related to medicine and mechanics to the image of a physician and to that of a mechanic (El Haj, 2017). Results demonstrated higher destination memory for consistent facts (e.g., facts concerning mechanics that were told to the mechanic) than for inconsistent facts (e.g., facts concerning mechanics that were told to the physician). These findings demonstrate how a sender may rely on stereotypical information attributed to the recipient when deciding to whom facts have been told previously.

The sender’s personality may also influence destination memory, especially if he/she is an extrovert. In a study on extroversion, participants performed a typical destination memory test and were asked to answer a questionnaire regarding extroversion (e.g., “I see myself as someone who is talkative/has an assertive personality”) (El Haj et al., 2021). Results demonstrated a significant positive correlation between extroversion and destination memory. According to this study, extroverts demonstrate high destination memory as they typically value social communication, public sharing and processing of social information.

The self may also influence destination memory. This effect was investigated in a study in which younger adults and older adults were asked to tell self-related information (e.g., “I like Chinese food”) and semantic information (e.g., “the moon is smaller than the sun”) to pictures of celebrities (El Haj et al., 2022). Results demonstrated higher destination memory for self-related information than for semantic information in both older adults and younger adults. These findings demonstrate high destination retention for self-related information, in other words, how individual may draw on self-related information to improve memory and social interactions in everyday life.

In brief, beyond the recipient’s influence, destination memory can be influenced by features related to the sender, especially by theory of mind which mediates his/her ability to process and retain the destination.

## 4. Discussion

**Figure 1:**
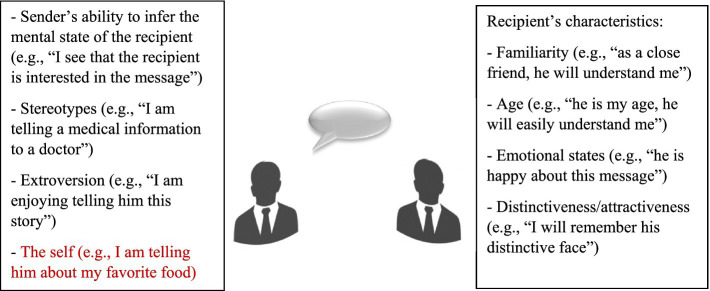
How we retain destination during a conversation When sending a message, the sender can associate the information with the mental/affective state of the sender or with stereotypes related to the recipient. The sender’s extroversion, and the self, may also facilitate retention. For better retention, the sender may also rely on the recipient’s characteristics.

In our daily lives, we constantly send information to friends, colleagues, family members and even to strangers. The ability to associate specific information with an interlocutor (i.e., destination memory) is essential for communicative efficacy and daily interactions with others. As demonstrated in this review, destination memory involves both the recipient of the information and its sender, as it can be influenced by factors related to both. As sketched in Figure 1, destination memory relies on the sender’s ability to infer the cognitive/affective state of the recipient and to attribute the output message to a stereotype related to the recipient. Extrovert senders may also retain the destination more easily, as they typically value social communication, public sharing and processing of social information. The self-value of the information may also influence destination memory. Destination memory also relies on features related to the recipient, such as familiarity, age, emotional states, distinctiveness and attractiveness.

In addition to synthesizing the literature about destination memory into a comprehensive framework and offering a “user guide” about how it works in everyday life interactions, the present review aims at providing directions for future studies. So far, research has mainly assessed destination memory using laboratory designs involving associations between information and pictures depicting faces. While this laboratory-based research is essential to control for the studied variables, one caveat is that it does not capture destination memory faithfully enough to mirror how it occurs in everyday life. Future research may could build on a study by Fischer et al. (2015), who offered a laboratory design that mimicked everyday life situations. Participants were asked to complete phrases in dyads and later remember to whom these sentences had been told. Future studies may also benefit from progress in virtual reality to create better controlled social environments allowing enhanced assessment of destination memory. Ideally, virtual reality designs could be coupled with behavioral/neurological assessments of factors that may influence destination memory, as highlighted in this review. Especially that little is known about the physiological and neuroanatomical correlates of destination memory. While promising, research on this topic stills is in its infancy (Mugikura et al., 2016; Iliadou et al., 2019; Li and Nie, 2021; Kladi et al., 2022). Another suggestion for future research is assessing a core, but neglected, sender’s characteristics, namely, whether some people would demonstrate highly superior destination memory. This suggestion is based on research focusing on individual differences that fall at some extremes of the “remembering” dimension, such as the differences between highly superior autobiographical memory and severely deficient autobiographical memory (Parker et al., 2006; Palombo et al., 2015, 2018; Santangelo et al., 2018, 2020).

Future research could also attempt to situate destination memory in a wider framework. It would be interesting to understand how it may improve communication and cooperation between senders and recipients. Destination memory can be beneficial for senders as it enables them to retain the context in which a message was encoded (e.g., not only to whom the message was relayed, but also the cognitive/affective state of the recipient during the communication). Such retention may help the sender establish communication with others and even be assertive about the occurrence of a given event (e.g., “I am sure that I asked that colleague about the data because he was very excited to assist me with it”). It would thus be interesting to assess how destination memory guides our interactions and our memory of them. In doing so, the recipient’s feedback should be carefully observed. During communication, we observe and evaluate the recipient’s feedback to adjust the content of the output message. This feedback may also serve as a salient cue during destination retrieval. However, this feedback has been surprisingly neglected in research on destination memory.

In conclusion, the study of destination memory paves the way for research on how memory might be intimately associated with social cognition. Owing to its social nature, destination memory improves social interaction with our environment. Thanks to it, we are able to attribute information to its appropriate recipient, avoiding the sharing of information with an inappropriate person or the development of inaccurate expectations of people with whom we interact.

## Author contributions

The author confirms being the sole contributor to this work and has reviewed and approved it for publication.

## Conflict of interest

The author declares that the research was conducted in the absence of any commercial or financial relationships that could be construed as a potential conflict of interest.

## Publisher’s note

All claims expressed in this article are solely those of the authors and do not necessarily represent those of their affiliated organizations, or those of the publisher, the editors and the reviewers. Any product that may be evaluated in this article, or claim that may be made by its manufacturer, is not guaranteed or endorsed by the publisher.
